# Effect of Uneven Electrostatic Forces on the Dynamic Characteristics of Capacitive Hemispherical Resonator Gyroscopes

**DOI:** 10.3390/s19061291

**Published:** 2019-03-14

**Authors:** Zeyuan Xu, Guoxing Yi, Meng Joo Er, Chao Huang

**Affiliations:** 1 Department of Control Science and Engineering, Harbin Institute of Technology, Harbin 150001, China; huangchao198311@126.com; 2 School of Electrical and Electronic Engineering, Nanyang Technological University, Singapore 639798, Singapore; emjer@ntu.edu.sg

**Keywords:** hemispherical resonator gyroscope, uneven electrostatic forces, capacitance gap, influence mechanism, dynamic characteristics, output error

## Abstract

The hemispherical resonator gyroscope (HRG) is a typical capacitive Coriolis vibratory gyroscope whose performance is inevitably influenced by the uneven electrostatic forces caused by the uneven excitation capacitance gap between the resonator and outer base. First, the mechanism of uneven electrostatic forces due to the significantly uneven capacitance gap in that the non-uniformity of the electrostatic forces can cause irregular deformation of the resonator and further affect the performance and precision of the HRG, was analyzed. According to the analyzed influence mechanism, the dynamic output error model of the HRG was established. In this work, the effect of the first four harmonics of the uneven capacitance gap on the HRG was investigated. It turns out that the zero bias and output error, caused by the first harmonic that dominates mainly the amplitude of the uneven capacitance gap, increase approximately linearly with the increase of the amplitude, and periodically vary with the increase of the phase. The effect of the other three harmonics follows the same law, but their amplitudes are one order of magnitude smaller than that of the first one, thus their effects on the HRG can be neglected. The effect of uneven electrostatic forces caused by the first harmonic on the scale factor is that its nonlinearity increases approximately linearly with the increase of the harmonic amplitude, which was analyzed in depth. Considering comprehensively the zero bias, the modification rate of output error, and scale factor nonlinearity, the tolerance towards the uneven excitation capacitance gap was obtained.

## 1. Introduction

Vibratory gyroscopes have drawn tremendous attention for their benefits of high precision, and high reliability, long life, and stable physical and chemistry properties, etc. The HRG has been used in many applications: oil borehole exploration, aircraft navigation, communication satellite stabilization, and deep space exploration, etc. Especially in the field of deep space exploration, HRG has been involved in a large number of asteroid exploration missions due to its legendary reliability and long life. From 1997 to 2017, the Cassini–Huygens probe equipped with HRG was in space for nearly two decades. The navigation data of the Cassini over nearly 20 years proves the excellent performance, high precision and long life of the HRG [1]. Therefore, it is worthwhile to investigate the HRG.

The resonator of the HRG is almost perfectly hemispherical and axisymmetric so as to exhibit excellent characteristics in terms of balancing, vibration characteristics, and damping isotropies, which are controlled through electrostatic forces thanks to electrodes located in the immediate proximity of the resonator [2,3]. The electrostatic detecting and exciting technique of the hemispherical resonator is achieved using an electrode to establish the electrostatic field. Electrostatic forces are produced by the piezoelectric effect that is applied in the field of current measurement and voltage control thanks to its high sensitivity [4,5]. When the resonator vibrates, the equivalent gap is changed so that the micro-current is detected and amplified to obtain the vibration signal of the resonator by the sensing circuit [6]. Therefore, the small error of capacitance gap will affect the electrostatic forces.

The error sources of the HRG mainly concentrate on two aspects which are the imperfect resonator and the manufacturing error of the electrodes. On the one hand, at present, many researchers are paying much attention to structure design optimization [7,8], controlling the manufacturing errors [9,10], and the imperfections of the materials [11,12] of the resonator. On the other hand, due to the limited precision of the manufacturing process, manufacturing errors of electrodes are inevitable. The distinct manufacturing errors of electrodes, such as alignment errors of electrodes [13], geometric form error of electrodes, and uneven capacitance gap, bring adverse effects to the HRG. The problem of parasitic capacitance caused by the manufacturing error of the electrodes is effectively controlled using two means, to increase the critical value of parasitic feed-through capacitance and increase the polarization voltage [14]. In practice, numerous error sources exist in the resonator, but the dominant error sources in state-of-the-art capacitive gyroscope is caused by the electrostatic forces used to drive and detect the response of the resonator [3,15]. Previous works have shown that the nonlinear electrostatic forces have effects on the mode coupling [15,16]. It was also found that the capacitance gap distance was a dominant factor for the initial capacitance, and affected the driving voltage and sensitivity [17]. In addition, a reasonable control method was proposed to suppress the effect of coupling between control loops caused by the electrode errors on the HRG [18,19,20,21]. Furthermore, a dynamic model that captures the effects of variation of electrostatic forces, elastic deformation, and residual stress on the mechanical performance was developed to analyze the influence mechanism [22,23,24].

The aim of this paper was to develop and apply an analytical model to quantify and understand the effect of the uneven electrostatic forces, caused by an uneven capacitance gap, on the dynamic response in the HRG.

## 2. Gyroscope Description and Theoretical Analysis

### 2.1. Gyroscope Structure

**Figure 1:**
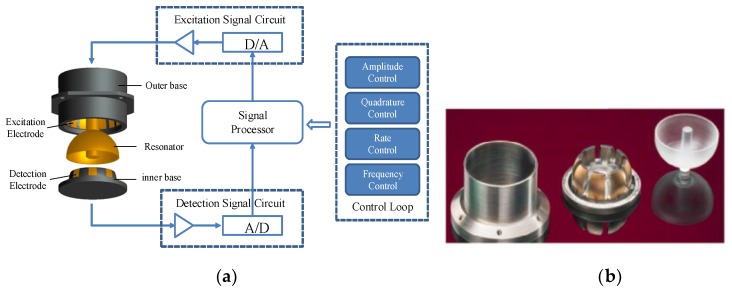
The basic structure of the HRG. (**a**) Simple structure diagram of the HRG; (**b**) The three main components (the resonator, inner base, and outer base).

The basic structure of the HRG with the force-to-rebalance mode consists of a metalized resonator, an outer base on which are evenly distributed 16 excitation electrodes, an inner base on which is evenly distributed 8 detection electrodes, and external circuits that collect the detection signal and generate the excitation signal, as in Figure 1.

Assuming that the quadrature drift of the standing wave is not considered, the excitation electrodes on the outer base together with the excitation electrode on the surface of the resonator form a series of excitation electrostatic capacitances that control the amplitude and phase of the standing wave. Because of the deformation of the resonator, detection capacitances are used to measure the changes of the capacitance to obtain the detection information.

### 2.2. Theoretical Analysis

#### 2.2.1. Uneven Electrostatic Forces

**Figure 2:**
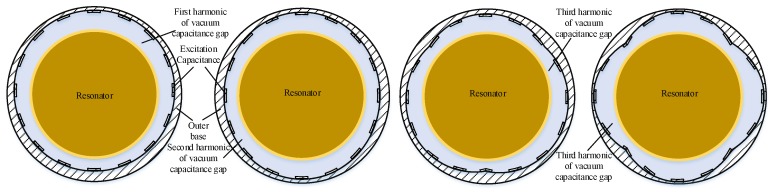
The structure diagram of first four harmonics of the uneven excitation capacitance gap.

It can be seen that under normal operating conditions the resonator vibrates with constant amplitude, which causes the electrode gap to vary by a small amount and ensures that the system operates within a second-order resonant state [15]. However, under external environmental factors and electrode errors, the electrostatic forces can vary unevenly due to a significantly uneven capacitance gap, which causes irregular deformation of the resonator and irregular in-plane motion of the resonator. In this paper, the first four harmonics of the uneven excitation capacitance gap were investigated in depth to obtain the effect of the uneven electrostatic forces. It is generally assumed that the capacitance gap *d* is given as follows in Figure 2.

(1) $$d=d_{0}+e_{1}\cos(\varphi +\phi_{1})+e_{2}\cos(\varphi +\phi_{2})+e_{3}\cos(\varphi +\phi_{3})+e_{4}\cos(\varphi +\phi_{4})$$

where $d_{0}$ is the mean value of the gaps, $e_{1}$, $e_{2}$, $e_{3}$, and $e_{4}$ are respectively the amplitudes of the 1st–4th harmonics of the uneven capacitance gaps, $\varphi_{1}$, $\varphi_{2}$, $\varphi_{3}$, and $\varphi_{4}$ are respectively the initial phases of the 1st–4th harmonics.

The exciting AC voltage of which the frequency is half the resonant frequency acts on the excitation electrodes to control the electrostatic forces driving the resonator to vibrate.

(2) $$V(\theta ,\varphi ,t)=\{\begin{array}{cc} V_{0}\cos\frac{\omega_{2}t}{2} & \left[\theta_{et},\theta_{eb}],[\varphi_{el},\varphi_{er}\right]\\ 0 & other \end{array}$$

where $V_{0}$ is the amplitude of the exciting voltage, $\omega_{2}$ is the resonant angular frequency, $\theta_{eb}$, $\theta_{et}$, $\varphi_{el}$ and $\varphi_{er}$ are respectively the top, bottom, left, and right boundary of the electrodes.

Comparing the size of capacitance gap (the micron level) with the size of radius of the resonator (the millimeter level), it is assumed that the spherical excitation capacitance can be simplified as the plate capacitance. The electrode plates of any charged capacitor are attracted (or repelled) to each other, so there are electrostatic forces between the excitation electrode and the resonator. Ignoring the boundary effect of the excitation electrodes, the electrostatic forces (surface force) acting on the resonator are given by

(3) $$f_{g}=\{\begin{array}{cc} -\frac{\varepsilon_{0}V^{2}}{2d^{2}} & \left[\theta_{et},\theta_{eb}],[\varphi_{el},\varphi_{er}\right]\\ 0 & other \end{array}$$

where $\varepsilon_{0}$ is the vacuum dielectric constant.

Equations (1)–(2) are substituted into Equation (3) to obtain the equation for the uneven electrostatic forces between the electrodes, which shows the dynamic relationship between the electrostatic forces and the uneven capacitance gaps.

(4) $$f_{g}=\frac{-\varepsilon_{0}V_{0}{}^{2}}{2d^{2}}\cos^{2}\frac{\omega_{2}t}{2}=\frac{-\varepsilon_{0}V_{0}{}^{2}}{2d^{2}}\frac{\cos\omega_{2}t+1}{2}=\frac{-\varepsilon_{0}V_{0}{}^{2}\cos\omega_{2}t}{4d^{2}}+\frac{-\varepsilon_{0}V_{0}{}^{2}}{4d^{2}}$$

The DC components of the exciting AC voltage are filtered out by the band-pass filter (BPF) to obtain the wanted signal. Thus, ignoring the constant component of the electrostatic forces, Equation (4) is simplified as follows:

(5) $$f_{g}=\{\begin{array}{cc} \frac{-\varepsilon_{0}V_{0}{}^{2}\cos\omega_{2}t}{4{\left(d_{0}+e_{1}\cos(\varphi +\varphi_{1})+e_{2}\cos(\varphi +\varphi_{2})+e_{3}\cos(\varphi +\varphi_{3})+e_{4}\cos(\varphi +\varphi_{4})\right)}^{2}} & \begin{array}{l} \left[\theta_{et},\theta_{eb}\right]\\ \left[\varphi_{el},\varphi_{er}\right] \end{array}\\ 0 & \mathrm{other} \end{array}$$

#### 2.2.2. Resonator Deformation

**Figure 3:**
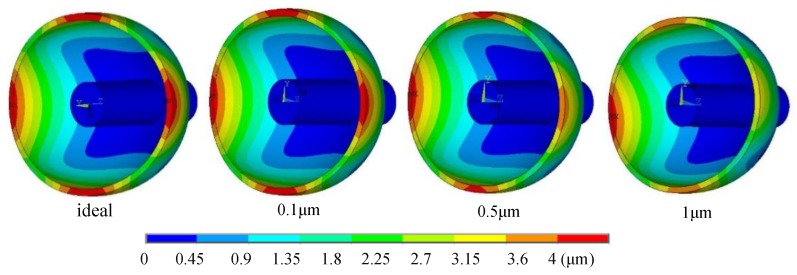
The selected simulation results of resonator deformation caused by the different amplitudes of the first harmonic of uneven capacitance gap.

The output error of the HRG is caused by the deformation of the resonator which is caused by the displacement of the center of mass and the uneven electrostatic forces used to maintain the vibration of the resonator. The uneven capacitance gaps can result in a change of electric field (that is uneven electrostatic forces) [15,21,25]. Thus, the uneven electrostatic forces will affect the output of the HRG. The selected simulation results of the resonator deformation caused by the excitation electrodes with different uneven capacitance gaps are obtained by finite element simulation that can intuitively and precisely describe the deformation characteristics of the resonator [26]. This demonstrated that the deformation of the resonator varies with the change of the capacitance gap. It is seen from Figure 3 that the vibration mode of the resonator with the first harmonic of the uneven capacitance gap is an irregular ellipse, and the deformation increases with the increase of the harmonic amplitude.

The wave amplitude is inversely proportional to the capacitance gap, that is, the bigger the uneven capacitance gap is, the smaller the wave amplitude is, and the more irregular the resonator deformation is. Because under the same exciting voltage, the bigger the uneven capacitance gap is, the weaker the electric field in the capacitor is, and the more uneven and smaller the electrostatic forces are, which leads to a smaller wave amplitude and greater irregular deformation.

**Figure 4:**
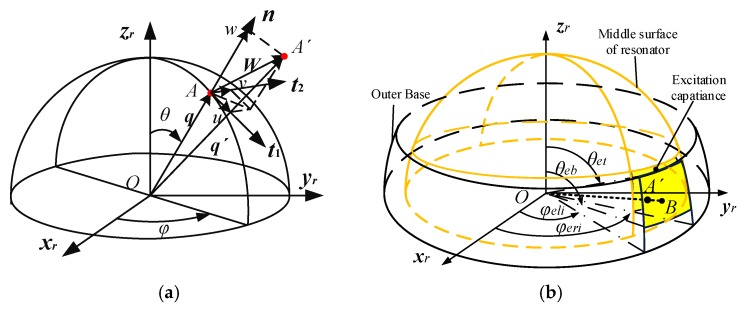
Structure diagram of the resonator: (**a**) The deformation of the resonator; (**b**) The excitation capacitance driving the resonator.

To analyze further the deformation characteristics of the resonator, the resonator was simplified to a hemispherical shell, which makes the shape of the excitation electrode plates on the outer base become spherical fan-shaped, as shown in Figure 4.

As shown in Figure 4, the point $A^{\prime }$ on the middle surface of the resonator corresponds to the point *B* on the excitation electrode of the outer base after the deformation, which builds the relationship between the radius of the excitation electrode *R_f_*, the radius of the middle surface of the resonator *R_d_*, the half thickness of the shell h/2, and the uneven capacitance gap *d*.

(6) $$d=R_{f}-R_{d}-h/2$$

It is necessary to establish the deformation equation of the resonator to obtain *R_d_*. The point *A* on the middle surface is deformed and moved to the point $A^{\prime }$, which obtains the deformation equation on the radius vector (***OA***), the radius vector (***OA*′**), and the displacement vector (***W***) after deformation, shown as follows in Equation (7).

(7) $$OA^{\prime }=\mathrm{OA}+W$$

In the resonator coordinate system ($Ox_{r}y_{r}z_{r}$), Equation (7) is described as

(8) $$R_{d}\left[\begin{array}{c} \sin\hat{\theta }\cos\hat{\varphi }\\ \sin\hat{\theta }\sin\hat{\varphi }\\ \cos\hat{\theta } \end{array}\right]=R\left[\begin{array}{c} \sin\theta \cos\varphi \\ \sin\theta \sin\varphi \\ \cos\theta \end{array}\right]+\left[\begin{array}{ccc} \cos\theta \cos\varphi & -\sin\varphi & \sin\theta \cos\varphi \\ \cos\theta \sin\varphi & \cos\varphi & \sin\theta \sin\varphi \\ -\sin\theta & 0 & \cos\theta \end{array}\right]\left[\begin{array}{c} u_{2}\\ v_{2}\\ w_{2} \end{array}\right]$$

where $u_{2}$, $v_{2}$, $w_{2}$ are the displacement components of the resonator in the local coordinate system ($t_{1}t_{2}n$). $\hat{\theta }$ and $\hat{\varphi }$ are the known ranges of the excitation electrodes, $\hat{\theta }\in [\theta_{et},\theta_{eb}]$, $\hat{\varphi }\in [\varphi_{el},\varphi_{er}]$. θ and φ are the unknown ranges of the excitation electrodes before deformation.

When the resonator enters into the steady resonant state, the vibration displacement equation of each mass point in the resonator can be expressed as follows [25,27].

(9) $$\begin{array}{l} u_{2}=U_{2}(p(t)\cos2\varphi +q(t)sin2\varphi ),\\ v_{2}=V_{2}(p(t)\sin2\varphi -q(t)\cos2\varphi ),\\ w_{2}=W_{2}(p(t)\cos2\varphi +q(t)\sin2\varphi ). \end{array}$$

where *p*(*t*) and *q*(*t*) are the time-dependent variables to be determined. *U*_2_, *V*_2,_ and *W*_2_ are the Rayleigh–Ritz functions [25,27].

(10) $$p(t)=a\sin\omega_{2}t,\;q(t)=b\sin\omega_{2}t$$

(11) $$U_{2}=V_{2}=\sin\theta \tan^{2}\theta /2,\;W_{2}=-(2+\cos\theta )\tan^{2}\theta /2$$

Further

(12) $$\begin{array}{l} u_{2}=A_{2}U_{2}\cos2(\varphi -\vartheta )\sin\omega_{2}t,\\ v_{2}=A_{2}V_{2}\sin2(\varphi -\vartheta )\sin\omega_{2}t,\\ w_{2}=A_{2}W_{2}\cos2(\varphi -\vartheta )\sin\omega_{2}t. \end{array}$$

(13) $$A_{2}=\sqrt{a^{2}+b^{2}},\;\tan2\vartheta =b/a$$

where *A*_2_ and *ϑ* are respectively the wave amplitude and vibration angle of the resonant vibration, *a* and *b* are the values related to the wave amplitude.

## 3. Dynamic Model

### 3.1. Dynamic Characteristics of Uneven Electrostatic Forces

**Figure 5:**
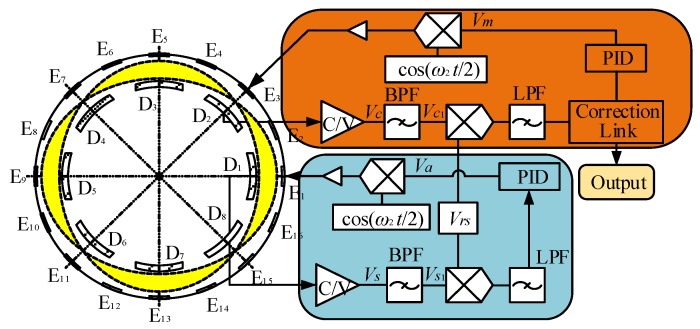
The structure diagram of the feedback control system for HRG with single-electrode mode.

An analysis method for the influence of feedback control on the dynamic characteristics of the piezoelectric HRG was developed to hold the standing wave in the initial position by phase feedback determining the angular rate, which demonstrates that the resonator could be balanced by the phase feedback used by the piezoelectric electrodes generating the electrostatic forces [5,20,21,28,29,30]. In force-to-rebalance mode, the measured angular rate is proportional to the voltages applied to the rate control loop. Electronic circuits may be used to measure the changes of these capacitances to determine the amplitude and phase of the standing wave. The different excitation voltages are applied to the excitation electrodes. The most basic form of feedback control system for the HRG is shown in Figure 5, which uses two excitation electrodes that are respectively E_1_ located at the 0° electrode axis and E_3_ located at the 45° electrode axis. E_1_ is used for driving the resonator and stabilizing the amplitude, and E_3_ is used for force feedback.

The motion equations of the resonator provide many insights into the dynamics of the resonator and show that the functions of the control system are required to effectively operate the resonator as an angular rate sensor. The analysis of the control problem begins with the motion equations of the resonator describing the dynamics of the resonator with the electrode error including the uneven electrostatic forces. In [31], a new method of treating the nonlinear electrostatic forces is presented using the Galerkin method to calculate the capacitance variation of the electrostatically actuated microplates. In the case of known dynamic equations and analytical solutions of the resonator, the motion equations of the resonator excited by the uneven electrostatic forces can be obtained based on the Bubnov–Galerkin method [27].

(14) $$\begin{array}{l} m_{0}\ddot{p}(t)+c_{0}\xi \dot{p}(t)-2b_{0}\Omega \dot{q}(t)+c_{0}p(t)=-(f_{a}S_{ca}+f_{m}S_{cm})\cos\omega_{2}t\\ m_{0}\ddot{q}(t)+c_{0}\xi \dot{q}(t)+2b_{0}\Omega \dot{p}(t)+c_{0}q(t)=-(f_{a}S_{sa}+f_{a}S_{sm})\cos\omega_{2}t \end{array}$$

where $V_{a}$ and $V_{m}$ are respectively the amplitude voltage and force feedback voltage. $f_{a}$ and $f_{m}$ characterize the amplitudes of the electrostatic forces. $S_{ca}$ is the amplitude excitation coefficient, $S_{sm}$ is the force feedback excitation coefficient, $S_{sa}$ and $S_{cm}$ are the coupling excitation coefficients.

(15) $$f_{a}=\varepsilon_{0}R^{4}V_{a}{}^{2}/4,f_{m}=\varepsilon_{0}R^{4}V_{m}{}^{2}/4$$

(16) $$\begin{array}{l} \begin{array}{c} S_{ci}=\int_{\varphi_{eli}}^{\varphi_{eri}}\int_{\theta_{eti}}^{\theta_{ebi}}\frac{W_{2}(\theta )\sin\theta \cos2\varphi }{{\left(d_{0}+e_{1}\cos(\varphi +\varphi_{1})+e_{2}\cos(\varphi +\varphi_{2})+e_{3}\cos(\varphi +\varphi_{3})+e_{4}\cos(\varphi +\varphi_{4})\right)}^{2}}d\theta d\varphi \\ S_{si}=\int_{\varphi_{eli}}^{\varphi_{eri}}\int_{\theta_{eti}}^{\theta_{ebi}}\frac{W_{2}(\theta )\sin\theta \sin2\varphi }{{\left(d_{0}+e_{1}\cos(\varphi +\varphi_{1})+e_{2}\cos(\varphi +\varphi_{2})+e_{3}\cos(\varphi +\varphi_{3})+e_{4}\cos(\varphi +\varphi_{4})\right)}^{2}}d\theta d\varphi \end{array}\\ \left(i=1,2,\cdots ,16\right) \end{array}$$

(17) $$K=b_{0}/2m_{0}$$

(18) $$m_{0}=-\pi \rho hR^{4}\int_{0}^{\pi /2}(U_{2}^{2}+V_{2}^{2}+W_{2}^{2})\sin\theta d\theta$$

(19) $$b_{0}=2\pi \rho hR^{4}\int_{0}^{\pi /2}V_{2}(W_{2}\sin\theta +U_{2}\cos\theta )\sin\theta d\theta$$

(20) $$c_{0}=\pi \int_{0}^{\pi /2}(\Gamma_{U}U_{2}+\Gamma_{V}V_{2}+\Gamma_{W}W_{3})\sin\theta d\theta$$

(21) $$\begin{array}{l} \Gamma_{U}=U_{2}\left(\frac{D+2D_{1}}{\sin^{2}\theta }+\mu D-D-\frac{N+4N_{1}}{\sin^{2}\theta }R^{2}+NR^{2}-\mu NR^{2}\right)+U_{2}^{\prime }\left(NR^{2}-D\right)\frac{\cos\theta }{\sin\theta }\\ -U_{2}^{\prime \prime }\left(D-NR^{2}\right)+V_{2}\left(D-NR^{2}-N_{1}R^{2}\right)\frac{4\cos\theta }{\sin^{2}\theta }-V_{2}^{\prime }\left(\frac{2\mu D+D_{1}}{\sin\theta }-2R^{2}\frac{N_{1}+\mu N}{\sin\theta }\right)\\ +W_{2}\frac{4\mu D\cos\theta +D\cos^{3}\theta +4D_{1}\cos\theta }{\sin^{3}\theta }+W_{2}\left(\frac{2D\cos\theta -\mu D\cos\theta }{\sin\theta }-NR^{2}\frac{\cos\theta }{\sin\theta }\right)+W_{2}^{\prime \prime \prime }D\\ -W_{2}\mu NR^{2}\frac{\cos\theta }{\sin\theta }+W_{2}^{\prime }\left(\frac{-4D_{1}-4\mu D-D\cos^{2}\theta -\mu D\sin^{2}\theta }{\sin^{2}\theta }+NR^{2}+\mu NR^{2}\right)+W_{2}^{\prime \prime }\frac{D\cos\theta }{\sin\theta } \end{array}$$

(22) $$\begin{array}{l} \Gamma_{V}=U_{2}^{\prime }\left(\frac{2\mu D-D_{1}}{\sin\theta }-2\frac{N_{1}+\mu N}{\sin\theta }R^{2}\right)-V_{2}\left(\frac{D_{1}\cos2\theta -8D}{2\sin^{2}\theta }+\frac{4N+N_{1}\cos2\theta }{\sin^{2}\theta }R^{2}\right)\\ +V_{2}^{\prime }\left(\frac{D_{1}}{2}+N_{1}R^{2}\right)\frac{\cos\theta }{\sin\theta }+V_{2}^{\prime \prime }\left(\frac{D_{1}}{2}+N_{1}R^{2}\right)+W_{2}\left(\frac{2D}{\sin^{3}\theta }+\frac{4D_{1}}{\sin\theta }\right)\\ -W_{2}\frac{4\mu N+4N}{\sin\theta }R^{2}-W_{2}^{\prime }\frac{2D\cos\theta }{\sin^{2}\theta }+W_{2}^{\prime \prime }\frac{2D_{1}-2\mu D}{\sin\theta } \end{array}$$

(23) $$\begin{array}{l} \Gamma_{W}=U_{2}^{\prime }\left(-\frac{4D_{1}+4\mu D+D+\mu D\sin^{2}\theta }{\sin^{2}\theta }+NR^{2}+\mu NR^{2}\right)+U_{2}^{\prime \prime }\frac{2D\cos\theta }{\sin\theta }+U_{2}^{\prime \prime \prime }D-V_{2}^{\prime }\frac{2D\cos\theta }{\sin^{2}\theta }\\ +V_{2}^{\prime \prime }\frac{2D_{1}+2\mu D}{\sin\theta }+W_{2}\frac{4(1+\mu )D(1+\cos^{2}\theta )-16D\sin\theta +8D_{1}}{\sin^{4}\theta }-2W_{2}(1+\mu )NR^{2}-W_{2}^{\left(4\right)}D\\ -W_{2}^{\prime }\frac{\cos\theta }{\sin\theta }\left(\frac{8D_{1}+8\mu D+D\cos^{2}\theta }{\sin^{2}\theta }+2D-\mu D\right)+W_{2}^{\prime \prime }\left(\frac{8\mu D+8D_{1}+D}{\sin^{2}\theta }+\mu D\right)-W_{2}^{\prime \prime \prime }\frac{2D\cos\theta }{\sin\theta } \end{array}$$

where R is the radius of middle surface, h is the thickness of shell, E is the Young’s modulus, μ is the Poisson ratio, $N=\frac{Eh}{1-\mu^{2}}$, $N_{1}=\frac{Eh}{2(1+\mu )}$, $D=\frac{Eh^{3}}{12(1-\mu^{2})}$, $D_{1}=\frac{Eh^{3}}{12(1+\mu )}$.

### 3.2. Output Error Model

It is concluded that p(t) and q(t) satisfy the second-order vibration displacement function. Substituting Equation (10) into Equation (14), yields

(24) $$\begin{array}{l} \omega_{2}^{3}\xi_{2}a-4K\Omega b\omega_{2}=-(f_{a}S_{ca}+f_{m}S_{cm})\\ \omega_{2}^{3}\xi_{2}b+4K\Omega a\omega_{2}=-(f_{a}S_{sa}+f_{m}S_{sm}) \end{array}$$

where

(25) $$S_{ca}=S_{c1},S_{cm}=S_{c3},S_{sa}=S_{s1},S_{sm}=S_{s3}.$$

Integrating Equations (13) and (24) further derived obtains the external angular rate Ω as follows

(26) $$\Omega =\frac{\omega_{2}^{2}\xi_{2}}{4K}\tan[\arctan\frac{f_{a}S_{ca}+f_{m}S_{cm}}{f_{a}S_{sa}+f_{m}S_{sm}}-2\vartheta ]$$

According to Equation (13), the vibration angle ϑ is calculated by using Equation (27).

(27) $$\vartheta =\frac{1}{2}\arctan(b/a)$$

Assuming that there is no structural error of electrodes, including the uneven excitation capacitance gap and shape error, in the HRG, there are no coupling excitation coefficients, which makes Equation (16) simplified as follows.

(28) $$S_{ci}=\int_{\varphi_{eli}}^{\varphi_{eri}}\int_{\theta_{eti}}^{\theta_{ebi}}\frac{W(\theta )\sin\theta \cos2\varphi }{d_{0}{}^{2}}d\theta d\varphi ,S_{si}=\int_{\varphi_{eli}}^{\varphi_{eri}}\int_{\theta_{eti}}^{\theta_{ebi}}\frac{W(\theta )\sin\theta \sin2\varphi }{d_{0}{}^{2}}d\theta d\varphi ,(i=1,2,\cdots ,16)$$

Thus Equation (26) is simplified as

(29) $$\Omega =\frac{\omega_{2}^{2}\xi_{2}}{4K}\tan\left(\arctan\frac{f_{m}}{f_{a}}-2\vartheta \right)$$

According to Equation (29), when the input angular rate is Ω, the vibration phase ϑ can be equal to zero by adjusting the ratio of *f_m_* and *f_a_*, that is, the force feedback electrode can hold the standing wave at the null position because the constant amplitude of the resonator signifies a constant electrostatic force. Thus, according to Equation (27), ϑ can be held at the null position to make *b* equal to zero, which results in Equation (24) becoming Equation (30).

(30) $$a=-\frac{S_{ca}f_{a}}{\omega_{2}^{3}\xi_{2}},\;\Omega =-\frac{S_{sm}f_{m}}{4Ka\omega_{2}}$$

Under ideal conditions, Equation (30) explains the control principle of the force-to-rebalance HRG from the perspective of the dynamics of the control system. In fact, due to the existence of the uneven capacitance gap, the uneven electrostatic forces have a bad effect on the control system of the HRG. In the working state of HRG, the amplitude control and force feedback control ensure that *a* equals constant, and *b* equals zero, which results in Equation (24) becoming the following equation.

(31) $$a=-(f_{a}S_{ca}+f_{m}S_{cm})/(\omega_{2}^{3}\xi_{2}),\;\Omega =-(f_{a}S_{sa}+f_{m}S_{sm})/(4Ka\omega_{2})$$

Further, Equation (31) is derived as

(32) $$\Omega =-\frac{f_{m}S_{sm}}{4Ka\omega_{2}}+\frac{f_{m}S_{cm}S_{sa}}{4Ka\omega_{2}S_{ca}}+\frac{\omega_{2}^{2}\xi_{2}S_{sa}}{4KS_{ca}}$$

From Equations (16) and (32), it can be seen that the uneven electrostatic forces can cause the changes of the amplitude excitation coefficients and force feedback excitation coefficients, as well as the existence of the coupling excitation coefficients. According to the working principle of the control system of capacitive HRG with the force-to-rebalance mode, this can adjust the control voltage to maintain constant amplitude and vibration mode angle, whereas the change of output control voltage directly represents the output error of the HRG.

## 4. Simulation Analysis

### 4.1. Effect of the First Four Harmonics

According to the fact that an equal capacitance gap between the adjacent electrodes will yield a better polarized electric field [32], it is concluded that uneven electrostatic forces due to an uneven capacitance gap will cause the change of the excitation coefficients, and further cause the zero bias and output error of the HRG.

**Figure 6:**
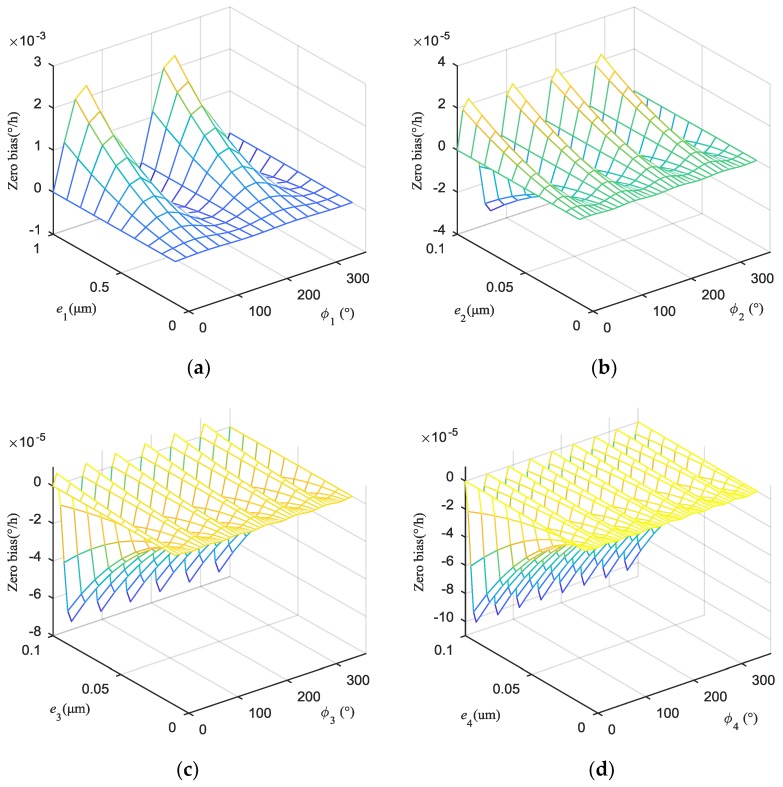
The zero biases caused by the first four harmonics of uneven capacitance gap are respectively: (**a**) Zero bias caused by the first harmonic; (**b**) Zero bias caused by the second harmonic; (**c**) Zero bias caused by the third harmonic; (**d**) Zero bias caused by the fourth harmonic.

The effect of the first four harmonics of the uneven excitation capacitance gap on the HRG was analyzed to obtain simulation results that depict the effect of the amplitude and phase of the first four harmonics, as shown in Figure 6. 

The amplitude of uneven capacitance gap is mainly dominated by the first harmonic component, which is caused by the machining technology of the circumferential gap. The amplitudes of the second harmonic, third harmonic, and fourth harmonic are one order of magnitude smaller than that of the first harmonic. The zero bias caused by the first harmonic increases approximately linearly with the increase of the amplitude, and varies periodically with the increase of the phase. When the amplitude of the first harmonic is 1 μm, the maximum of zero bias is 2.274 × 10^−3^°/h. The zero biases caused by the second harmonic, third harmonic, and fourth harmonic respectively exhibit periodic changes of four-period oscillation, six-period oscillation, and eight-period oscillation. Their maxima are respectively 2.274 × 10^−5^°/h, 7.563 × 10^−5^°/h and 1.03 × 10^−4^°/h which are smaller than that of the first harmonic, which can be neglected. For the same amplitude, the higher the harmonic order in the uneven capacitance gap is, the more uneven the electrostatic forces are, and the larger the zero bias is.

**Figure 7:**
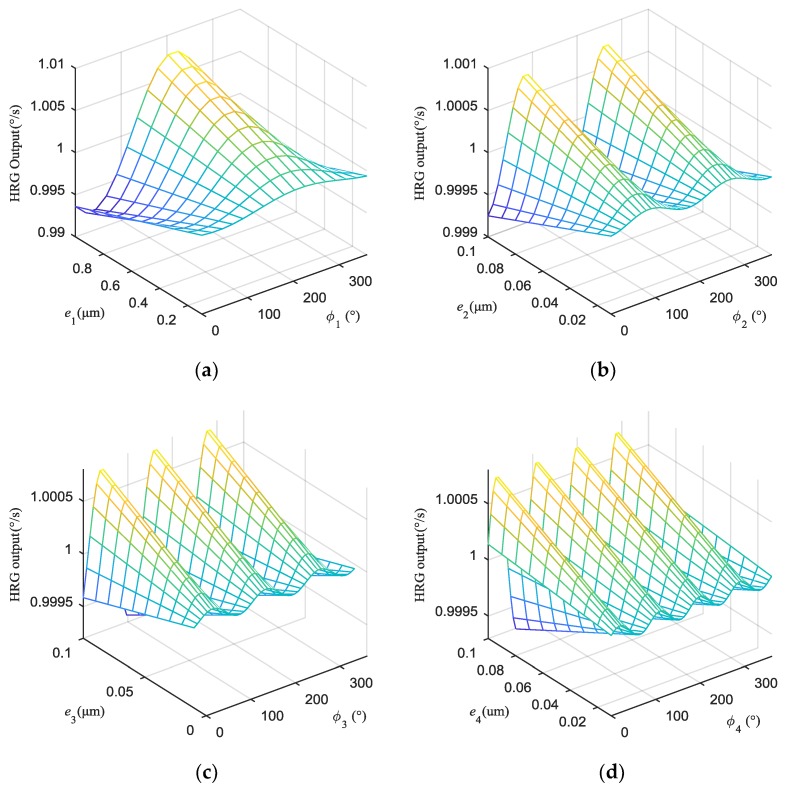
The output errors caused by the first four harmonics of uneven capacitance gap under 1°/s are respectively: (**a**) Output error caused by the first harmonic; (**b**) Output error caused by the second harmonic; (**c**) Output error caused by the third harmonic; (**d**) Output error caused by the fourth harmonic.

The output error caused by the uneven capacitance gap increases approximately linearly with the increase of the amplitude and varies periodically with the change of the phase in Figure 7. For example, when the input angular rate is 1°/s, the maxima of the output errors caused by the second harmonic, third harmonic, and fourth harmonic are respectively 7.506 × 10^−4^°/s, 7.318 × 10^−4^°/s and 7.06 × 10^−4^°/s that is an order of magnitude smaller than that of the first harmonic (7.659 × 10^−3^°/s), which indicates that the output error of HRG is mainly affected by the uneven electrostatic forces caused by the first harmonic. It is seen that the effect of uneven electrostatic forces on the output of the HRG cannot be neglected, in particular the uneven electrostatic forces caused by the first harmonic.

**Figure 8:**
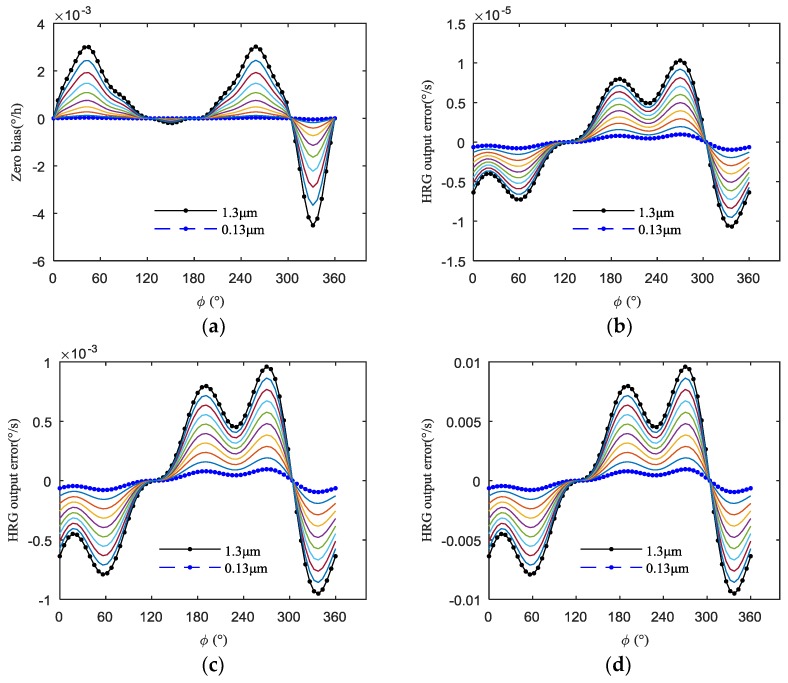
The HRG output error caused by the first four harmonics of uneven capacitance gap under the different input angular rates are respectively: (**a**) Zero bias caused by the first four harmonics; (**b**) Output error (Input: 10^−3^°/s); (**c**) Output error (Input: 0.1°/s); (**d**) Output error (Input: 1°/s).

The outputs of the HRG are obtained by setting different input angular rates (± 0.001°/s, ± 0.003°/s, ± 0.005°/s, ± 0.007°/s, ± 0.01°/s, ± 0.03°/s, ± 0.05°/s, ± 0.07°/s, ± 0.1°/s, ± 0.3°/s, ± 0.5°/s, ± 0.7°/s, ± 1°/s, ± 1.5°/s, ± 2°/s, ± 2.5°/s, ± 3°/s). Because the amplitude of the uneven capacitance gap is mainly dominated by the first harmonic, the amplitudes of the first harmonic are changed from 0.1 μm to 1 μm with an increment of 0.1 μm, and the amplitudes of the other three harmonics are changed from 0.01 μm to 0.1 μm with an increment of 0.01 μm. In Figure 8a, it is seen that the maximum of zero bias is 4.511 × 10^−3^°/h which is caused by the first four harmonics, which indicates that each harmonic component has a large degree of influence on the zero bias. However, it is seen from Figure 8b–d, that the output error of the angular rate is approximately periodically varied with the change of the phase, which is mainly dominated by the first harmonic component. In Figure 8d, when the angular rate is 1°/s, the maximum of output error is 0.0095°/s. In the single-electrode control mode, the tolerance range of excitation capacitance gap is given, which is, zero bias controlled within the range of 4.124 × 10^−4^°/h, and the output error controlled within the range of 0.2885%, while the amplitude of the uneven capacitance gap should be less than 0.33 μm.

**Figure 9:**
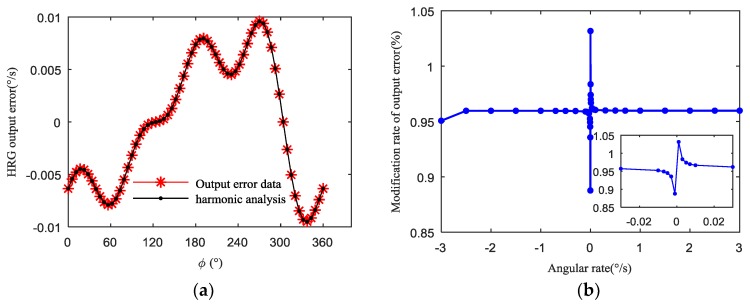
The HRG output error caused by 1.3 μm–270° uneven capacitance gap in the angular rate 1°/s: (**a**) Harmonic analysis of the HRG output; (**b**) The modification rate of output error of the HRG output.

In the case, when the amplitude of the uneven capacitance gap is 1.3 μm and the initial phase is 270°, the harmonic analysis and modification rate of output error for the HRG are respectively shown in Figure 9 and Table 1. It is seen that the uneven capacitance gap is mainly dominated by the first harmonic component, which is almost identical to the results of the first four harmonics separately analyzed. Moreover, the modification rate of the output error for the HRG increases sharply with the angular rate approaching 0, and the change of the modification rate of output error is nonlinear, basically. For the same uneven capacitance gap, the closer the angular rate is to zero (that is, the non-uniformity of the electrostatic forces is greater), the greater the modification rate of the output error is, which can affect the scale factor nonlinearity.

### 4.2. Effect of Uneven Electrostatic Forces

**Figure 10:**
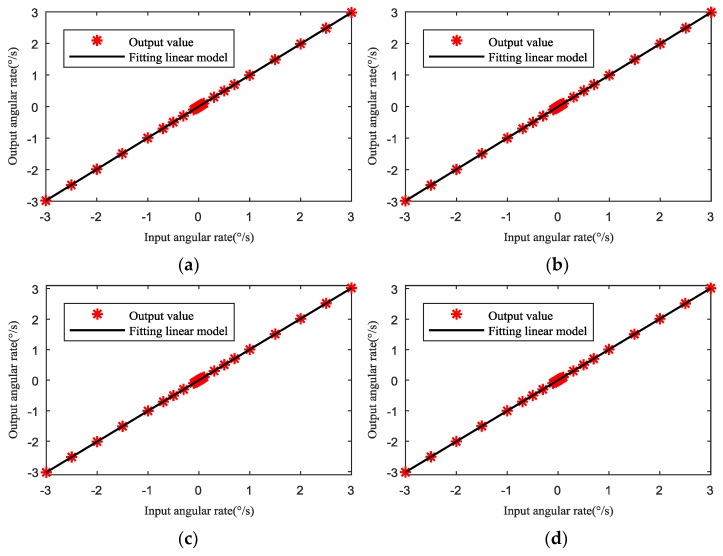
The fitting curves of the HRG output under the same harmonic amplitude (1 μm) and different initial phases (0°, 90°, 180°, and 270°) of uneven capacitance gap are respectively: (**a**) Initial phase 0°; (**b**) Initial phase 90°; (**c**) Initial phase 180°; (**d**) Initial phase 270°.

Because the uneven capacitance gap is mainly dominated by the first harmonic, the influence of uneven electrostatic forces caused by the first harmonic on the scale factor was analyzed in depth. For the first harmonic, the selected results of the HRG output for 1 μm uneven capacitance gap are shown in Table 2 and Figure 10a. The results in Table 2 and Figure 10 show that the first harmonic in the uneven capacitance gap can cause the output error of the HRG, and also cause scale factor nonlinearity.

The linear model of the HRG input and output is

(33) $$\Omega_{Oi}=K_{1}\Omega_{Ii}+K_{0}+\varepsilon_{i}$$

where $K_{1}$ is the scale factor, $K_{0}$ is the fitting zero bias, $\varepsilon_{i}$ is the fitting residuals.

Fitting a straight line describes the input–output relationship:

(34) $${\hat{\Omega }}_{Oi}=K_{1}\Omega_{Ii}+K_{0}$$

where ${\hat{\Omega }}_{Oi}$ is the output of the HRG on fitting a straight line corresponding to the input angular rate $\Omega_{Ii}$.

**Figure 11:**
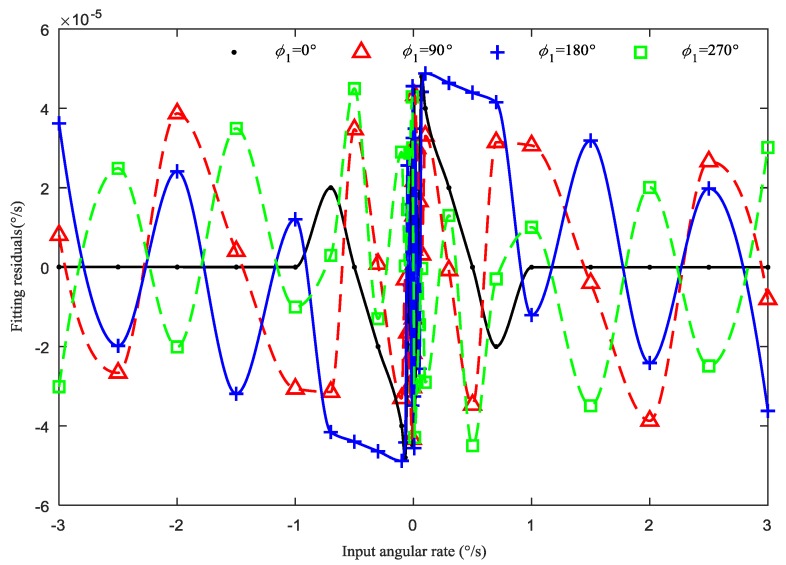
Fitting residuals of angular rate under 1 μm–0°, 90°, 180°, and 270° uneven capacitance gap.

In four cases of uneven capacitance gap (1 μm–0°, 1 μm–90°, 1 μm–180° and 1 μm–270°), the scale factor and fitting residuals are respectively shown in Table 3 and Figure 11. It is seen that, for the same amplitude, different initial phases of uneven capacitance gaps can cause different effects on the scale factor and fitting residuals. Thus, the different initial phases lead to the different uneven electrostatic forces, and further cause different scale factors and fitting residuals.

The results in Figure 10a–d show that the effects of the four different initial phases (0°, 90°, 180°, and 270°) on the scale factor are different under the same harmonic amplitude (1 μm). By comparison, it is seen that the fitting curves in the four cases are different, which can cause different scale factor nonlinearity.

The scale factor nonlinearity is, in the range of input angular rate, the ratio of the maximum fitting residual of the HRG output relative to the fitting line by using the least square method for the output of maximum input angular rate. The formula of the scale factor nonlinearity is shown as:

(35) $$\delta ={\left|\frac{{\hat{\Omega }}_{Oi}-\Omega_{Oi}}{\left|\Omega_{O\max}\right|}\right|}_{\max}$$

where δ is the scale factor nonlinearity, $\left|\Omega_{O\max}\right|$ is the maximum the output of input angular rate.

**Figure 12:**
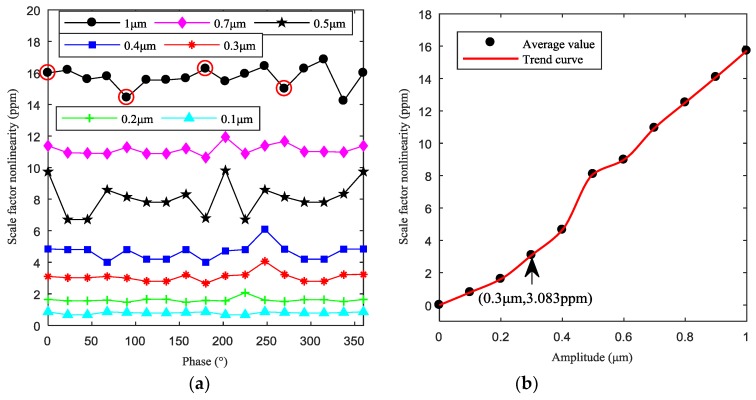
Scale factor nonlinearity: (**a**) Scale factor nonlinearities caused by different uneven capacitance gaps; (**b**) Effect of amplitudes of uneven capacitance gap on the average nonlinearity.

In the four cases, in the condition of same amplitude of uneven capacitance gap, different electrostatic forces caused by different initial phases lead to different scale factor nonlinearities (red round marker in Figure 12a), where their values are respectively 16 ppm, 14.44 ppm, 16.26 ppm, and 14.99 ppm. The difference of the nonlinearities can be a consequence of the nonlinear effects of the electrostatic forces. In Figure 12a, when the amplitude of the uneven capacitance gap is small *e*_1_ = 0.1 μm (as cyan triangle marker), the maximum of the nonlinearities is 0.8586 ppm (less than 1 ppm). When *e*_1_ equals 0.3 μm (as red star marker), the maximum of nonlinearities is 4.062 ppm. The main conclusion drawn from this section studies is that the bigger the amplitude of uneven capacitance gap, the more uneven are the electrostatic forces, and the bigger is the scale factor nonlinearity. The amplitude and initial phase of uneven capacitance gap codetermine the differences of the uneven electrostatic forces as well as the influence on the scale factor nonlinearity.

Figure 12b shows the modification trend of scale factor nonlinearities. It can be seen that, from 1 μm to 0.5 μm, the scale factor nonlinearity decreases approximately linearly with the decrease of harmonic amplitude. When the harmonic amplitude passes 0.5 μm, the scale factor nonlinearity firstly drops suddenly (from 0.5 μm to 0.3 μm), and then decreases slowly with the decrease of harmonic amplitude (from 0.3 μm to 0). This illustrates that 0.3 μm is the critical value of the uneven capacitance gap which is also the critical value of the change of the uneven electrostatic forces. 

## 5. Conclusions

This paper investigated the effect of uneven electrostatic forces caused by the uneven capacitance gap on the dynamic properties of the resonator in the HRG. The mathematical model was established to calculate the resonator deformation and further analyze the effect of uneven electrostatic forces on the zero bias and output error for the HRG. A finite element model for hemispherical resonator was established to demonstrate the accuracy of the mathematical model. It was demonstrated that the zero bias and output error increase approximately linearly with the increase of the amplitude of uneven capacitance gap and vary periodically with the increase of the phase under the same voltage. The output errors of the HRG are mainly dominated by the first harmonic of uneven capacitance gap. In the case of the same harmonic amplitude, the higher the harmonic order in the uneven capacitance gap is, the larger the zero bias is. The bigger the amplitude of the first harmonic is, the more uneven the electrostatic forces are, and the bigger the scale factor nonlinearity is. In the single-electrode control mode, the tolerance towards the uneven capacitance gap is obtained, that is, the zero bias being controlled within 4.124 × 10^−4^°/h, the modification rate of output error being controlled within 0.2885%, and the scale factor nonlinearity being controlled within 4 ppm, the amplitude of the uneven capacitive gap should be less than 0.3 µm. Therefore, it is advantageous to improve the machining and installation technology of the capacitive resonator for better performance of the HRG. 

## Figures and Tables

**Table 1:** Harmonic analysis results of the HRG output for 1.3 μm–270° uneven capacitance gap(1°/s).

Uneven Capacitance Gap	First Harmonic	Second Harmonic	Third Harmonic	Fourth Harmonic
Amplitude	0.0078°/s	0.0015°/s	0.0021°/s	0.0026°/s
Initial phase	123.82°	67.30°	11.39°	314.82°

**Table 2:** HRG output for 1 μm–0° uneven capacitance gap.

Input Angular Rate (°/s)	Output Angular Rate (°/s)	Input Angular Rate (°/s)	Output Angular Rate (°/s)	Input Angular Rate (°/s)	Output Angular Rate (°/s)	Input Angular Rate (°/s)	Output Angular Rate (°/s)
−3	−2.9808	−0.07	−0.0696	0.003	0.0030	0.5	0.4968
−2.5	−2.4840	−0.05	−0.0497	0.005	0.0050	0.7	0.6955
−2	−1.9872	−0.03	−0.0298	0.007	0.0070	1	0.9936
−1.5	−1.4904	−0.01	−0.0099	0.01	0.0099	1.5	1.4904
−1	−0.9936	−0.007	−0.0070	0.03	0.0298	2	1.9872
−0.7	−0.6955	−0.005	−0.0050	0.05	0.0497	2.5	2.4840
−0.5	−0.4968	−0.003	−0.0030	0.07	0.0696	3	2.9808
−0.3	−0.2981	−0.001	−0.0010	0.1	0.0994		
−0.1	−0.0994	0.001	0.0010	0.3	0.2981		

**Table 3:** The linear model of the HRG output.

First Harmonic	1 μm–0°	1 μm–90°	1 μm–180°	1 μm–270°
*K*_1_	0.9936	0.9957	1.0065	1.0043
*K*_2_	0	2.7756 × 10^−17^	1.3878 × 10^−17^	2.7756 × 10^−17^
